# Impact of Resistance Exercise and Nutritional Endorsement on physical performance in patients with GvHD (IRENE-G study) – design and rational of a randomized controlled trial

**DOI:** 10.1186/s12885-022-09497-1

**Published:** 2022-04-22

**Authors:** Janina Bujan Rivera, Rea Kühl, Ulrike Zech, Anne Hendricks, Thomas Luft, Peter Dreger, Birgit Friedmann-Bette, Theresa-Maria Betz, Joachim Wiskemann

**Affiliations:** 1 Division of Medical Oncology, National Center for Tumor Diseases (NCT), University Hospital Heidelberg, Im Neuenheimer Feld 460, 69120 Heidelberg, Germany; 2 Department of Internal Medicine I, University Hospital Heidelberg, Im Neuenheimer Feld 410, 69120 Heidelberg, Germany; 3 Department of Internal Medicine V, University Hospital Heidelberg, Im Neuenheimer Feld 410, 69120 Heidelberg, Germany; 4 Department of Internal Medicine VII, University Hospital Heidelberg, Im Neuenheimer Feld 410, 69120 Heidelberg, Germany; 5 Department of Internal Medicine III, University Hospital Heidelberg, Im Neuenheimer Feld 410, 69120 Heidelberg, Germany

**Keywords:** Cancer, Exercise, Graft-versus-host disease, Glucocorticoids, Resistance training, Nutritional endorsement, Allogeneic stem cell transplantation, Muscle atrophy, Physical performance, Malnutrition

## Abstract

**Background:**

Graft-versus-host disease (GvHD) remains a major complication and limitation to successful allogeneic hematopoietic stem cell transplantation. Treatment of GvHD is challenging due to its heterogeneous nature of presentation, with steroids remaining the established first-line treatment. Long-term doses of systemic corticosteroids have many well-known side-effects including muscle atrophy. Despite the fact that reports in non-cancer clinical populations treated with glucocorticoids demonstrated that resistance training can reverse atrophy and weakness, no RCT has evaluated the potential of resistance training on preventing the disease- and treatment-induced loss of skeletal muscle mass and function in GvHD patients yet. In this context, ensuring adequate nutrition is important as protein deprivation may accelerate the wasting process. As GvHD patients are commonly found to be malnourished, nutritional medical care should be considered when investigating the effect of exercise in GvHD patients. Therefore, the aim of the present “Impact of Resistance Exercise and Nutritional Endorsement on physical performance in patients with GvHD” – Study (IRENE-G) is to evaluate the effects of resistance exercise in combination with nutritional endorsement on physical, nutritional and patient-reported outcomes in GvHD patients.

**Methods:**

IRENE-G is a 24-week prospective interventional RCT. One hundred twelve participants will be randomly allocated (1:1) to one of two arms: resistance exercise and nutritional optimization (experimental) vs. nutritional optimization only (control). Participants in the experimental group will engage in a supervised, progressive moderate-to-high intensity resistance training that is consistent with exercise guidelines for cancer patients, while additionally receiving nutritional support/therapy. Subjects of the control group solely receive nutritional support/therapy based on individual needs. Participants will be assessed at baseline, at 8, 16, 24 weeks for physical performance and various physiological, nutritional and patient-reported outcomes. Follow-up will be 6 months after intervention completion.

**Discussion:**

To our knowledge, this will be the first RCT to assess and compare the effects of a resistance intervention supplemented by nutritional support/therapy against nutritional support only on various health-related outcomes in GvHD patients. The study will contribute to our understanding of the value of exercise and nutritional endorsement in counteracting the negative consequences of GvHD and its treatment.

**Trial registration:**

ClinicalTrials.gov: NCT05111834. Registered 8 November 2021 – Retrospectively registered.

## Background

### Allogeneic hematopoietic stem cell transplantation and Graft-versus-host disease

Allogeneic hematopoietic stem cell transplantation (allo HSCT) is a potentially curative treatment modality for various malignant and non-malignant diseases [1]. In 2019, almost 20,000 allo HSCT were performed in Europe [2]. A major barrier for successful allo HSCT represents the Graft-versus-host disease (GvHD), as it remains the main cause for transplant-related morbidity and mortality [3–10]. The incidence of GvHD ranges widely, depending on various patient- and transplant-related variables. Roughly, 30–50% of patients develop an acute form of GvHD [3, 11–13]. The aggregated cumulative incidence for the chronic form of GvHD is estimated to be 30–50% [14, 15]. Risk factors for both forms of GvHD include degree of human leukocyte antigen mismatch, older donor age and donor-recipient gender mismatch [16]. A GvHD may appear in many different ways and can manifest in multiple organs e.g. lung, liver, skin and gastrointestinal tract. Consequently, patients suffer from organ-specific symptoms such as diarrhea, nausea, vomiting, exanthema, shortness of breath. The clinical manifestation has implication for patients’ physical function, limiting a patient’s ability to carry out activities of daily living and subsequently reduces the quality of life [6, 17–22].

Impairments in the physical domain is a result of both the disease itself and its treatments. Currently, the first-line therapy for both acute and chronic GvHD consists of glucocorticoids alone or in combination with calcineurin inhibitors, with solely 40–50% of patients responding adequately [23, 24]. Unfortunately, usage of glucocorticoids is associated with a variety of side-effects, especially at higher doses and with longer duration of therapy, such as osteoporosis, osteonecrosis, diabetes and myopathy with weakness primarily found in the proximal lower muscles, with particularly the pelvic girdle muscles being involved [23, 25, 26].

In view of the poor treatment respond and the toxic effects of the GvHD therapy, new supportive strategies that will help maintain or even improve patients’ quality of life are needed. Such supportive therapies should particularly target the physical domain, hence, reducing impacts on activities of daily living resulting in the preservation of public participation and autonomy.

### Physical function and exercise in the cancer/HSCT setting

There is strong scientific evidence that exercise can ameliorate the detrimental effects of cancer and its treatment. These beneficial effects include increases in physical fitness (i.e. aerobic capacity and muscle strength) and quality of life, improvements/prevention of lymphedema symptoms, reductions in fatigue, anxiety and depressive symptoms [27]. Furthermore, a growing body of evidence suggests positive effects of exercise on cancer-specific as well as all-cause mortality [28]. Hence, experts recommend cancer patients to participate in moderate-intensity aerobic training for a minimum of 30 min on 3 days per week and additional resistance training for at least two times per week [27]. Benefits of exercise can also be observed in the HSCT setting. A 2021 systematic review [29] yielded that exercise interventions may be beneficial on physical functioning and quality of life in patients undergoing HSCT. The findings of the positive effects of exercise on HSCT patients are supported by another review which found beneficial effects for muscle strength and physical fitness [30]. Specifically in patients receiving an allo HSCT, randomized controlled studies [31–35] showed that exercise is capable of counteracting the negative consequences of cancer and its treatment and may improve survival [36]. Despite such benefits, however, to date there is no scientific evidence of the effects of a structural exercise intervention in a subgroup of GvHD patients, specifically not among patients treated with high-dose steroids. Pre-clinical findings in a chronic GvHD murine model under standard immunosuppressive therapy suggest beneficial effects of exercise on survival, clinical course of GvHD and on physical capacity in the exercising mice group compared to control animals. Moreover, the exercising mice showed lower TNF-α and IL-4 levels after 12 weeks post transplant, reflecting a weaker inflammatory state [37]. These findings give first insight on how exercise may affect the clinical and biological course of GvHD patients. Unfortunately, not only the GvHD per se deteriorates the patients’ health status but also the treatment with corticosteroids. A prospective study authored by Morishita et al. [38] showed that the cumulative corticosteroids dose is associated with weak handgrip and knee extension. This is in line with recent findings of a small single-arm cohort study by Ngo-Huang et al. [39], who investigated acute GvHD patients on high-dose steroids and their decline in objective functional tests. They found a significant association between cumulative corticosteroid dose and the following functional tests: 6 min walk test, hip flexors and knee flexors strength, manual muscle testing strength, 5 sit to stand test. In terms of the timing of the decline, weakness can be detected as early as day 14, suggesting that early supportive interventions are needed to mitigate these changes. Interestingly, Morishita et al. [38] found that physical therapy is positively associated with physical function, indicating that exercise may be capable of ameliorating the detrimental effects of GvHD and its treatment. Given the fact that exercise has shown promise in counteracting the negative consequences of allo HSCT and can be considered as safe and feasible [30], there is a strong rational that it might be capable of ameliorating the detrimental effects of a GvHD too.

### Nutritional interventions in the Cancer/HSCT Setting

In order to encounter the decrements in physical performance and further side-effects in GvHD patients, ensuring adequate nutrition is important as protein deprivation may accelerate the loss in skeletal muscle mass and function. The nutritional status of cancer patients can vary significantly throughout the cancer continuum, with malnutrition often occurring [40–42]. According to the European Society for Clinical Nutrition and Metabolism expert group, approximately 10–20% deaths of patients with cancer are attributed to malnutrition rather than to the disease itself [43]. Further, cancer- and treatment-related malnutrition has been shown to be associated with increased morbidity [44], altered quality of life, particularly in the physical functioning scale [45–48], psychosocial stress [49], increased hospital length of stay [41, 50, 51] and increased healthcare costs [51]. Hence, nutritional support is an important aspect of cancer care. Malnutrition often involves cachexia, a multifactorial syndrome which is characterized by loss of skeletal muscle mass with or without loss of fat mass that leads to progressive functional impairment [52]. This condition is often accompanied by anorexia, which is driven by disturbances of the central mechanisms controlling food intake, resulting in loss of body weight, alterations in body composition and decline in physical function [53]. Patients undergoing allo HSCT are at an increased risk for these conditions, due to the toxic effects of treatments and/or due to secondary complications such as infections or GvHD [53, 54]. Studies report that a considerable number of patients undergoing allo HSCT suffer from worsening in their nutritional status during hospitalization and after discharge [55, 56]. Particularly patients with GvHD are vulnerable to malnutrition, as symptoms related to the disease and its treatment are driving forces for this condition (e.g. nausea, vomiting, mucositis of the mucous membrane of the upper gastrointestinal tract) [55, 57]. A study authored by Bassim et al. [58] reported a prevalence of malnutrition among chronic GvHD patients of 29%, highlighting the need for nutritional support in this specific population. The authors also reported that malnourished chronic GvHD patients have poorer survival compared with those without malnutrition and that malnutrition is associated with gastrointestinal tract, lung and mouth manifestations and impaired functional status and quality of life [58]. An even higher incidence of malnutrition among GvHD patients has been found in a study by Jacobsohn et al. [59]. According to their analysis 43% of GvHD patients were considered as malnourished [59]. Despite the incidence and adverse effects of malnutrition, the condition is often overlooked. However, addressing malnutrition at an early stage is important to mitigate nutritional derangements as soon as possible and maintain skeletal muscle mass and physical performance.

Nutritional intervention approaches seem to be effective in counteracting malnutrition in cancer patients. A review by Lee et al. [60] found that nutritional counselling with or without oral nutrition supplements is associated with improvements in different aspects of nutritional status outcomes including weight gain and maintenance, BMI and patient-generated subjective global assessment score in cancer patients.

### Combined exercise and nutritional support

Given nutrition’s foundational importance for various health-outcomes and for physical aspects, it is likely that a combination of an exercise and nutritional intervention will be of greater benefit than one intervention in isolation. Previous reviews which investigated the effects of combined nutritional and exercise interventions in incurable and colorectal cancer patients found improvements in multiple domains, including physical endurance [61, 62], suggesting that a combined approach may also be effective in other cancer populations. Despite the promising results of a combined exercise and nutrition program, a recent review by Prins et al. [29] showed that, to date, there is no study using such an approach in the HSCT setting. The IRENE-G study (acronym for Impact of Resistance Exercise and Nutritional Endorsement on GvHD symptoms) addresses the research gap and aims to examine the effect of a progressive moderate-to-high intensity resistance exercise program (2x/week for a total duration of 24 weeks) in combination with nutritional optimization on physical performance in GvHD patients treated with high-dose steroid therapy. To the best of our knowledge, this is the first RCT that specifically focuses on GvHD patients. The results of the IRENE- G study will enhance our understanding of the effectiveness of exercise interventions in combination with structured nutritional therapy measures for acute and chronic GvHD.

## Methods/design

### Study design

The IRENE-G study is a 24-week prospective, randomized, controlled, intervention trial with a two-armed parallel design and 1:1 allocation ratio of GvHD patients treated with systemic steroids.

The study examines and compares the effects of a supervised resistance exercise intervention and a nutritional optimization program (experimental group) against a nutritional optimization program only (control group) on physical performance in GvHD patients treated with steroids. The investigators hypothesize that both groups will benefit with regard to physical performance after 24-weeks, however, the authors assume that the effect will be significantly greater in the experimental group compared to the control group. Moreover, the investigators hypothesize that the experimental group will have lower levels of fatigue, less perceived GvHD symptom burden, a higher perceived quality of life, better muscle strength values and a higher vastus lateralis muscle volume, a better nutritional status as well as a better submaximal endurance capacity compared to the control group.

**Fig. 1:**
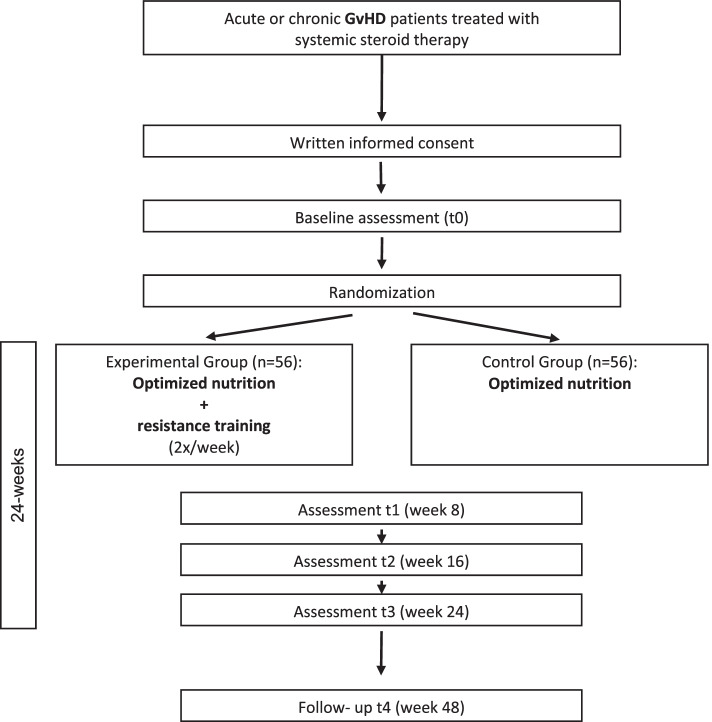
Study flow

The flow through the IRENE-G study is presented in Fig. 1. The research will be conducted at a single site (University Hospital Heidelberg, Germany). Upon written informed consent, eligible patients will be randomly assigned to the experimental or control group. Patients in the experimental group will take part in a 24-week supervised resistance exercise program and a nutritional optimization program. The moderate-to-high intensity resistance exercise sessions will be performed for a duration of 60 min two times a week. Patients in the control group receive nutritional optimization only. The interventions will start directly after the completion of the baseline measurements (t0). Further assessments will take place 8 weeks (t1), 16 weeks (t2), and 24 weeks (t3, primary endpoint assessment) after baseline assessment. A follow-up assessment (t4) is planned 48 weeks after baseline. All assessment points are embedded into medical care/ outpatient visits.

The study will be performed in accordance with the Declaration of Helsinki and has been approved by the Medical Ethic Committee of the University of Heidelberg (number S-433/2021). Any protocol amendment which may impact the study conduct (e.g. changes to outcomes or eligibility criteria) will be communicated to and needs approval of the ethic committee prior to implementation. The Study Protocol has undergone full external peer review by the funding body as part of the peer review process in the Funding Declaration. This study protocol is written in accordance with the SPIRIT guidelines. The study is registered at ClinicalTrials.gov (NCT05111834).

### Study outcomes

An overview of the outcome measures used in the IRENE-G study, including the time of data collection can be viewed in Table 1. The analysis metrics of interest are difference between the treatment arms throughout the study and change from baseline to t3.

**Table 1:** Study outcomes, instruments and assessment points

Outcomes	Instrument	t0	t1	t2	t3	t4
*Primary endpoint*						
Physical performance	Short Physical Performance Battery	x	x	x	x	x
*Secondary endpoints*						
Muscle strength	Hand-grip test, Hand-held Dynamometer	x	x	x	x	x
Muscle volume	Ultrasound	x			x	x
Submaximal endurance capacity	6 minutes walk test	x	x	x	x	x
Nutritional status	NRS 2002, hand- grip test, serum albumin, total protein levels	x	x	x	x	x
Quality of life	EORTC QLQ-C30/ HDC-29	x	x	x	x	x
Fatigue	EORTC QLQ-FA 12	x	x	x	x	x
GvHD Symptom burden	Modified 7-day Lee cGvHD Symptom Scale	x	x	x	x	x
Performance status	Karnofsky Performance Scale	x	x	x	x	x
*Others*						
Socio-demographic factors	Sex, age, marital status, family and living situation, education, employment status and smoking behavior	x			x	x
Physical activity (history)	Physical activity pre-diagnosis and during the intervention	x	x	x	x	x
Amnestic variables, medical history	Date of diagnosis, subtype of disease, remission status, conditioning regime, history of therapy, date of allo HSCT transplantation, HLA compatibility and comorbidities will be recorded from medical records	x				
Anthropometric data	Weight, height	Weekly				
Concomitant medication	Medical log	At each medical outpatient visit				
GvHD course	Physician rating	At each medical outpatient visit				
Adverse events	Medical records, reports of the exercise therapists and/or patient	Surveillance throughout the study				
Adherence to the exercise intervention	Self-reported and objective measures (e.g. attendance, exercise log, target intensity) of the intervention arm only	Surveillance throughout the study				

### Primary outcome measure

#### Physical performance

The short physical performance battery (SPPB) [63] will be used to evaluate lower extremity physical performance status. The SPPB has been adopted in various studies, especially in those of aging [63–66], and has shown to be associated with mobility disability, future institutionalization and mortality [64, 67, 68]. The test battery comprises three objective timed tests: 1) gait speed test, 2) five chair-stands test, 3) balance test. The gait speed test measures the time needed for walking a distance of four meter at normal pace. The chair-stands test measures the time needed to complete five sit to stands as quickly as possible without using upper extremity assistance. In the balance test, the participants are advised to hold their balance in three standing positions with eyes open: feet side-by-side position, feet in a semi-tandem position and feet in tandem stand. Each stance will be demonstrated to the participant by the study investigator before participant attempts. A score from 0 (being unable to complete the task) to 4 (highest level of performance) will be assigned to each performance. By summing the three individual scores, a total physical performance score will be created, with higher scores indicating better lower body function [63]. As these tasks mimic daily activities, this is an important outcome measure for patients with GvHD. The instrument has been used successfully in a collective of hemato-oncological patients [69] and in previous trials investigating physical performance in cancer patients [70–72].

### Secondary outcome measures

#### Muscle strength

A hand-held dynamometer (device from CITEC©, Netherlands), which is a recommended instrument to measure muscular strength in cancer patients [73], will be applied to assess maximal voluntary isometric muscle contraction in Newton meters in six muscle groups (knee and elbow flexors and extensors, hip flexors and abductors). Results of a study by Knols et al. [74] indicate that there is acceptable reliability for evaluating muscle strength with a hand-held dynamometer in patients with hematological malignancies. Strength test will be conducted in standardized test positions according to Bohannon [75]. The patient will be instructed to perform maximum contraction during dynamometry measures. Muscle groups will be assessed bilaterally for their isometric power. The maximum force applied within 3 s of effort will be recorded. For each muscle group, measurement will be carried out in triplicate. The best value will be used in the analysis. In addition, a hand-grip strength test will be performed using a digital hand dynamometer RFM (DHD-1) of the brand saehan. Measurement will be taken from both hands in a sitting position with the elbow extended and arm parallel to, but not touching, the side of the body. After the demonstration of the technique, the investigator will instruct the patient to perform maximum contraction during the measures. For each hand, the patient will perform three valid sets. The maximum value (in kg) within 3 s of effort will be recorded. Both procedures are well-established and have been used in hematological cancer populations [33, 35, 38, 76, 77].

#### Submaximal endurance capacity

The 6 min walk test will be used to determine the submaximal endurance capacity [78]. The test will be performed according to the American Thoracic Society protocol [79]. Briefly, patients will be instructed to walk as fast as possible on a flat, easy-to-walk track for 6 min. The total distance walked in 6 min under maximal effort conditions will be measured. Further, heart rate and O_2_-saturation will be assessed before, during and after the test and the individual perceived exhaustion will be measured using the Borg-Scale [80]. The 6 min walk test has been reported to have good psychometric properties in a variety of areas including cancer patients [81]. It is a simple and safe test which has previously been used in allo HSCT patients [35, 38, 76]. The formula by Enright et al. [82] will be used for reference.

#### Muscle volume

Ultrasound measurement will be performed to determine the thickness and cross-sectional area of both vastus lateralis muscles as well as subcutaneous adipose tissue thickness. Muscle ultrasound measurement is a non-invasive and safe measurement. Both in elderly people and in younger populations, studies showed a good reliability of muscle ultrasound and a good validity compared with MRI, CT or DEXA yet [83, 84]. Reliability and validity of our standardized ultrasound examination protocol to quantify vastus lateralis muscle was shown in a recently conducted study [85].

Patients will be sited in supine position with a soft roll below the hollow of their knees. They will be instructed to relax their leg muscles during the whole examination. The distance between the upper patella pol and the spina iliaca anterior superior will be measured and three measurements sites will be marked on the skin in the middle, the lower and upper third of the vastus lateralis muscle (VL) using a flexible tape measure. On each measurements site, the medial and lateral edges of VL belly will be identified by ultrasound. Then again, a flexible tape-measure will be used to connect the medial and lateral edge of VL by drawing a line and to mark the mid-point of this distance. This point will be defined the reference point for each measurements site. On each reference point, three fixed images of VL will be taken. For measuring muscle cross sectional area, the extended field of view software (LOGIQview, GE Healthcare GmbH) will be used reconstructing panoramic images of VL belly.

#### Quality of life

Patients’ quality of life will be assessed using the validated 30-item self-assessment questionnaire of the European Organisation for Research and Treatment of Cancer (EORTC QLQ-C30, version 3.0). It is composed of five functional multi-item scales (physical, role, emotional, cognitive, and social function), three symptom multi-item scales (fatigue, pain, nausea/vomiting), one global health status multi-item scale, and six single items for remaining symptoms [86]. The EORTC QLQ-C30 will be supplemented by the EORTC QLQ-HDC 29 module. This 29-item questionnaire assesses the quality of life during and after high-dose chemotherapy and stem cell transplantation [87]. It consists of 6 multi-item scales and 8 single-item questions. Scoring will be performed in accordance with the EORTC scoring manual [88].

#### Fatigue

The score of the EORTC QLQ-FA 12 will be used to assess physical, emotional and cognitive fatigue in participants. The EORTC QLQ-FA 12 is a self-assessment questionnaire of the European Organisation for Research and Treatment of Cancer and an internationally validated phase IV module. It is composed of 12 questions, 10 unidirectional and 2 criteria variables which measure the extent to which fatigue interferes with daily activities and social life [89]. Scores will be derived according to the EORTC scoring manual.

#### GvHD symptom burden

The original Lee chronic GvHD Symptom scale has been recommended for use in clinical practice and trials by the 2005, 2014 and the 2020 National Institutes of Health Consensus Conference [90–92]. In this study, the modified 7-day Lee Chronic-versus-Host Disease Symptom Scale (mLSS) will be used to capture the GvHD-specific symptom burden. The mLSS is a 28 item, 7-domain symptom scale (skin, eyes, mouth, lung, nutrition, energy and psych) that takes 2 min to complete. Patients will report their level of symptom bother over a period of 7 days on a 5-point Likert scale. Scoring will be performed according to the scoring algorithm. The mLSS is a reliable and valid instrument for evaluating GvHD symptoms [93]. According to authors of the mLSS, a 5 to 6 change in the summary score suggests a clinically meaningful difference in patient’s symptomatology [93].

#### Nutritional status

Nutritional status will be screened and adopted regulatory and performed individualized with multiple tools. First, the Nutritional Risk Screening (NRS 2002), a validated screening tool for detecting malnutrition in hospitalized settings, is used [94]. NRS includes a pre-screening, containing four yes/no-questions about body mass index, weight loss, food intake and disease severity. By answering at least one question with yes, the main screening will be performed, specifying and ranking the questions from pre-screening and also considering patients age. A total score > 3 implements a risk for malnutrition and requires further nutritional adjustment. Scores < 3 require regular repetition.

Based on NRS an individual nutritional counselling according to nutritional guidelines of the German Society for Nutritional Medicine and the European Society for Clinical Nutrition and Metabolism is performed. Weekly counseling includes monitoring of relevant blood parameters (e.g. serum albumin and total protein) and ensuring the daily calorie (25-30 kcal/kg bodyweight/d) and protein needs (1.2–1.5 g protein/kg body weight/d) are fulfilled. Finally, hand grip strength (see section muscle strength), known as a marker of muscle function and good nutritional state is performed regularly [95, 96].

#### Performance status

The Karnofsky performance scale will be applied to assess functional status of a patient. The Karnofsky performance scale is a 11-point rating scale, which ranges from normal functioning (100%) to dead (0%) [97]. The scale is widely used due to its psychometric properties and recommended in clinical assessments [91, 98].

### Study sample

Patients are considered eligible for study participation if they meet the following inclusion criteria:

1. Present acute or chronic GvHD which is currently treated with systemic steroids
2. Age ≥ 18 years
3. Sufficient mastery of the German language in order to understand and follow the study protocol
4. Willing/able to train twice a week live-online supervised by sport therapists, or at exercise facilities at the National Center for Tumor Diseases or at certified training facilities
5. Willing to take part in the scheduled assessments
6. Ability to consent and give written informed consent

Exclusion criteria are:

1. Any physical or mental condition that would hamper the performance or interfere with the exercise program or the completion of the study procedures, e.g.
  - Heart insufficiency > NYHA III or uncertain arrhythmia
  - Uncontrolled hypertension
  - Severe renal dysfunction (GFR < 30%, Creatinine> 3 mg/dl)
  - Reduced standing or walking ability
  - Insufficient hematological capacity (either hemoglobin value below 8 g/dl or thrombocytes below 30.000/μL)
  - Any other comorbidities that preclude participation in the exercise programs

### Sample size

To our knowledge no supervised, progressive moderate-to-high intensity resistance exercise study in a GvHD population treated with systemic steroids with the primary endpoint physical performance measured via the SPPB exists, as of yet. Consequently, the effect size for the primary outcome measure is unknown. The sample size calculation for the present exploratory project is based on a conservative estimation, using a mean standardized effect size of 0.5. To detect a corresponding effect with ANCOVA at a significance level of 5% with a power of 80%, 42 participants per group are required. Based on unpublished data of a previous study of ours, we expect a drop-out rate of 15% and a mortality rate of 10%. Consequently, 56 subjects per group need to be enrolled.

### Recruitment and randomization

The goal is to recruit 112 patients within an inclusion period of 2.5 years. Recruitment will take place at the University Hospital in Heidelberg - Section stem cell transplantation. All potentially eligible patients seen by their treating hematologist will be invited to participate in the study and will be referred to the case management. The case manager will provide eligible patients with the study flyer and the patient information. Further, they will obtain patients’ consent to be contacted by the study investigators via phone. On a daily basis, study investigators will collect the consent forms of referred patients and contact them by phone to provide further information on the study, review interest and eligibility. In case interested patients contact study investigators directly, they will be informed about the main aspects of the study and initially screened for eligibility. Patients who are still interested in participating in the study after the initial phone contact are invited to have an in-person discussion. During that discussion, participants have the opportunity to ask remaining questions about the research and express concerns. Before written informed consent will be obtained, the study investigators seek verbal assurance that the patient understands the study aim, its interventions and procedures and that the participation is entirely voluntary. Study investigators will inform the patient that his/her approval may be withdrawn at any time, for any reason, and without disadvantages for further medical care. Patients who are still willing to participate then need to provide written informed consent. No financial incentives for subjects who take part in the research study are offered. Upon written informed consent, an appointment for baseline assessment will be scheduled. Patients who are not willing to participate are asked to provide the reasons for non-participation.

After the completion of the baseline measurements, patients will be randomly assigned to one of the two arms with a 1:1 allocation, using REDCap (Research Electronic Data Capture) randomization module (automated assignment system) [99, 100]. To avoid imbalance between the arms, block randomization procedure is applied. Stratification factors for the randomization include intensity of conditioning for allo HSCT (reduced-intensity vs myeloablative), haploidentical transplantation (yes vs. no) and type of GvHD (acute vs. chronic). The allocation table has been generated with R [101] by an project-independent employee of the research department based on the given requirements.

Allocation concealment will be ensured, as the randomization module will not release the code until the patient has been recruited into the trial and has completed the baseline assessment. After assignment to one of the arms, neither participants nor staff can be blinded to allocation due to the nature of interventions and staffs’ direct involvement within the intervention program (e.g. training organization, phone calls about participants’ wellbeing).

### Data collection and management

Participants will be required to attend the University Hospital Heidelberg on five occasions. All study visits will be planned, if possible, with routine care visits. All assessments will last between 1.5–2 h. Participants will be advised to wear comfortable shoes and clothes and to arrive hydrated to the study visit. Assessments will be done by study investigators, who have been extensively trained. Moreover, a number of pilot tests were performed prior to the study. To maintain high methodological quality, internal inspections of the methods will be carried out. Data will be collected and managed using REDCap electronic data capture tools hosted at the University Hospital Heidelberg. REDCap is a secure web application for building and managing databases developed at Vanderbilt University and launched in 2004 [99, 100]. Data will be entered digitally throughout each assessment using a mobile tablet. Only study investigators will have access to the REDCap project and will be able to do entries. The principal investigator and the study coordinator will have the right to unlock entries after assessment completion to do major adjustments. To assure high data quality, background validation has been installed for the majority of variables. Remaining variables will be checked for logic by study coordinator.

When participant attends the assessment, he/she will be asked to complete a survey using a mobile tablet. The survey includes sociodemographic questions, questions on fatigue, GvHD symptoms and on quality of life. Patients will receive immediate feedback when implausible values have been given, hence, responds can be adjusted. If assistance is needed, the study investigators will administer participants via interview. In case of tablet rejection, participant can respond on a paper-based questionnaire. After completion, physical assessments will be conducted, followed by the nutritional assessment. Patients will be contacted via telephone for the following assessment appointments.

Reasons for drop-out throughout the study will be recorded where possible. No further data will be collected in patients who withdrew from the study.

### Data monitoring

A Data monitoring committee will not be included in this project as the trial involves behavioral interventions with known and especially minimal risks. Hence, no periodic benefit-risk assessment is needed. No independent auditing of study conduct is planned.

### Confidentiality

Confidentiality (with regard to the Federal Data Protection Act) of all patient-related data will be ensured, as all information will be pseudonymously (encrypted) and stored securely at the study site in areas with limited access. Moreover, access within the project team will be the minimum required for data analysis and quality control. A separate log relating original patient data with its respective, encrypted data will be created and appropriately secured by password; only authorized study personal will be granted access to this file.

### Interventions

Both study interventions (nutritional optimization as well as exercise training) will be delivered for a duration of 24 weeks followed by a 6 month follow-up phase. For both arms there are arrangements for ancillary or post-trial care. No special arrangements for compensation for those who suffered harm resulting from trial participation are taken. A decision to discontinue an individual’s intervention will be made jointly within the investigator team if there is concern that the intervention is causing harm. The overall study will be discontinued if the ethic committee decides to terminate the study. During the trial, no specific concomitant care or interventions are prohibited.

### Nutritional intervention

The nutritional status of participants of the control and of the experimental group will be examined at t0 using the NRS 2002, measurements of the hand grip dynamometer and medical/laboratory data which were collected during the latest clinical practice (e.g. weight, blood parameters like total protein, albumin, c-reactive protein, electrolytes as sodium, potassium, magnesium, phosphate, kidney and liver values, glucose, triglyceride). Based on participants individual needs and/or changes in general condition/nutritional status, nutritional recommendations are given. If malnutrition or a risk for malnutrition is detected, first step will always be oral supplementation, e.g. with protein supplements or oral nutritional supplements. In severe cases a nutritional therapy with e.g. enteral or parental infusion therapy will be initiated. Study participants will be contacted weekly to adapt nutritional therapy.

Throughout the study, nutritional recommendations and therapy will be adapted based on the risk of malnutrition (NRS 2002 score ≥ 3, low hand grip strength (men < 30 kg; women < 20 kg) defined as at least two standard deviations below the mean of the norm value, low serum albumin (< 30 g/l) and total protein (< 60 g/l)).

**Fig. 2:**
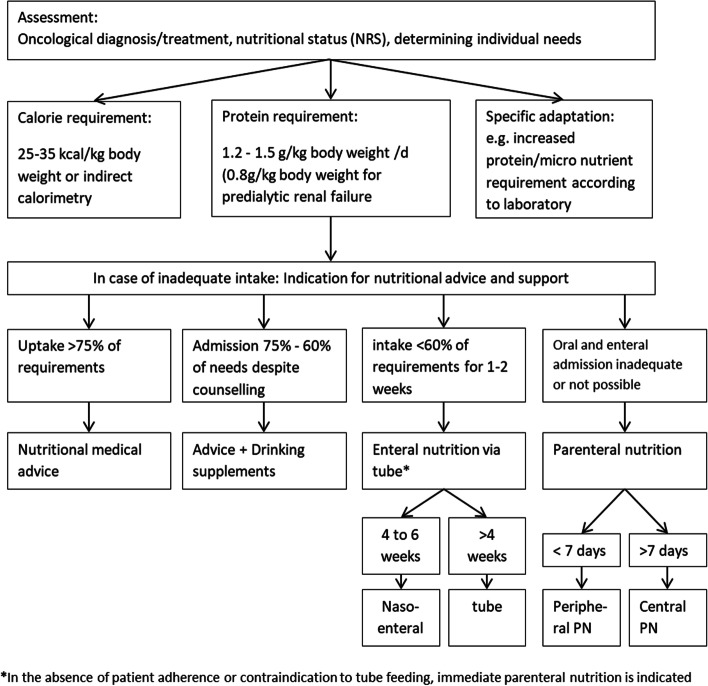
Flow algorithm optimized nutritional medical care according to Virizuela et al. [102]

The main nutritional intervention is based on the scheme by Virizuela et al. [102] shown in Fig. 2. If the energy and protein level is below 75% of the previously defined requirement for a maximum of 7 days, a re-evaluation takes place and oral nutritional supplements may be used. If there is an indication for additional action such as enteral or parenteral nutrition, this will also be initiated. If enteral feeding via tube is refused by the patient or if the use of a tube is contraindicated from a medical point of view, parenteral nutrition will be started in consultation with the study patient and the physician. In addition to macronutrients, nutritional therapy always ensures an adequate supply of micronutrients. If study patients of both groups (experimental and control) still need nutritional therapy after intervention period, it will be ensured as part of the usual medical care system.

### Exercise intervention

Participants of the experimental group receive, in addition to nutritional endorsement, a supervised progressive moderate-to-high-intensity resistance training program. Depending on their medical condition and Covid-19 precautions and immune status patients have the opportunity to train live-online or at our exercise facility at the National Center for Tumor Diseases (NCT) or at certified training facilities near their home provided by the network OnkoAktiv.

Participants will be provided with an exercise intervention booklet. The booklet, written in plain language, contains educational information about physical exercise and cancer, information about the exercise intervention process, weekly physical activity logs and a catalogue with illustrated exercises (~ 15) for strengthening the muscles. Moreover, patients will receive stretch bands with different levels of tension. After the handover, the booklet will be discussed verbally with the participant by the study investigators, to ensure comprehension.

Regardless of the training location, participants will be supervised by an experienced exercise therapist or physiotherapist. Introductory training sessions will be scheduled before the start of the intervention program in order to screen the patients and to adapt the program in case of physical limitations. Each training session begins with a low-intensity warm-up (5–10 min stretching or mobility exercises), followed by the resistance training regime (45 min), and a cool-down (5–10 min) which will include stretching of major muscle groups. Online exercise session will be offered in small groups on weekday afternoons in order to ensure that participants who have medical appointments or other commitments are able to participate.

Exercise training is scheduled 2 times a week (separated by at least 48 h) for a total duration of 60 min. The program targets all major muscle groups, however, emphasis will be put on the lower extremities, as they are primarily affected by the GvHD treatment. The focus of the resistance training will be on muscle hypertrophy. The progressive program for participants who will train at facility centers comprises of 6–8 machine-based exercises, each performed in two sets, 12 repetitions at 60–80% of one repetition maximum (1-RM). For participants who train online, the individual training load will be defined during the introductory training session with the aim of choosing a load which to perform two times 12 repetitions. Patients will be coached in correct techniques for each exercise. Resting time between sets will be 1–2 min, and contractions will be performed at moderate velocities. The exercise intensity will be continuously adapted throughout the 24 weeks. The training is progressive in terms of weight increase to the next machine weight level (at least by 5%) after successfully completing two sets of an exercise with 12 repetitions in three consecutive exercise sessions. If deteriorations occur, the weights will be decreased, if two sets of an exercise within 12 repetitions in two consecutive exercise sessions cannot be attained. Participants will be contacted when 2 session in a row were not joined, without reasoning. Compliance with the exercise program will be determined via self-reported and objective measures such as attendance, exercise logs, target intensity.

Before and after each training session, participants will be asked to complete an exercise log. The physical activity log will be used for exercise monitoring and for adjusting. Moreover, it will be used as a measure of additional physical activity outside of the exercise intervention (such physical activity is not restricted or prohibited). Patients will be asked to report their perceived health status prior and after the training session as well as problem or symptom occurrence related to the exercise program. Further, the performed sets and repetition as well as the perceived exertion of each exercise and discomfort will be recorded. Every 4 weeks patients are instructed to drop off the logs during outpatient visit.

To promote retention patients will be contact weekly in the first 4 weeks and then every 2 weeks. Exercise sessions will temporarily be interrupted if severe pain, dizziness, nausea or other contraindication (thrombocytes ≤20.000/μl, hemoglobin ≤8.0 g/dl, fever (≥38,0 °C), severe infections, acute thrombosis or embolism, increased tendency to bleed) occur. When the study is complete, participants will be encourage to continue exercising in certified training facilities of the network OnkoAktiv or individually. Training protocol comply with the American College of Sports Medicine exercise guidelines for cancer survivors and with the American College of Sports Medicine recommendations for progressive resistance training for novice weightlifters and older adults for one to three sets at a weight that can be done/lifted for 8 to 12 repetitions (approximately 60–80% of 1-RM).

Participants assigned to the control group will be provided with the general physical activity recommendations for cancer survivors from the American College of Sports Medicine, which is compatible with health-related physical activity guidelines for the general population, due to ethical reasons, as evidence shows that physical activity during the recovery phase and in cancer survivors is beneficial for the overall health [30]. After completion of the study, the control subjects will be offered mediation to a certified training facility for cancer patients/survivors.

### Safety

Adverse event monitoring will begin after participants have been randomized and will last 24 weeks. All adverse events related to the intervention will be recorded and reported according to the standardized guidelines for reportable events. The grading will be done using the Common Terminology Criteria for Adverse Events by the US National Cancer Institute. Patients will be informed about contraindication for exercise training and will be advised to stop exercising when they feel pain or other symptoms.

### Intended statistical analysis

Data of the primary endpoint will be analyzed after study completion, no interim statistical analysis will be conducted. Descriptive statistics will be performed for sociodemographic and clinical variables measures at baseline for both arms. Absolute and relative frequencies will be used for categorical and the mean ± standard deviation (or median and range) will be used for continuous variables. In addition to descriptive evaluation procedures, inferential statistical methods will be used. The primary analysis will be based on an intention-to-treat principle. The authors will use ANCOVA to compare the SPPB changes from baseline to the end of the intervention between the two intervention arms. Standardized effect sizes with 95% confidence intervals will be calculated. For further exploratory analyses further model procedures (e.g. generalized estimating equation) and multiple regressions will be used. The significance level will be set at α = 0.05 (two-sided). Missing data will be dealt with using scoring manuals if present. When clinically and methodological reasonable, multiple imputation will be used. Adverse events will be evaluated descriptively based on the per-protocol population. Statistical analyses will be carried out in cooperation with the Institute for Medical Biometry at the University of Heidelberg. Access to the final dataset will be granted for the statistician and the principal investigators.

### Dissemination and data sharing policy

Independent of the magnitude or direction of the effect of resistance training and nutritional optimization on physical performance, the results of the primary analysis and secondary analyses will be disseminated. The results of the primary analysis will be addressed in one main publication. Due to the number of secondary outcomes, further publications are planned. Results of the study will be submitted to the ClinicalTrials.gov results database and communicated to the participants via written report. Additionally, upon request, participants may receive a report on their personal result written in plain language.

## Discussion

A GvHD and its treatment is associated with impairments in patient’s functional capacity and performance [21], as well as with nutritional derangements [58, 59] and psychological distress [103], all of which result in substantial deterioration in patients’ quality of life. As a consequence, patients with GvHD are at high need for supportive care interventions.

There is strong evidence that endurance and resistance training in allo HSCT patients is safe and elicits beneficial effects [27, 30]. Further, nutritional intervention approaches seem to be effective in counteracting malnutrition in cancer patients [60]. Despite the promising results of the interventions in isolation, to date, there is no RCT using such an approach in patients with GvHD.

The IRENE-G study aims to bridge the research gap and enhance our understanding of the effectiveness of exercise in combination with structured nutritional therapy measures on various health-related outcomes in patients with GvHD.

The key strength of this study is its design. IRENE-G uses a randomized controlled study design to determine the effect of progressive resistance exercise on physical performance and further outcomes in patients with GvHD. This methodological approach represents the gold standard and has the advantage of reducing bias, also relating to confounding factors. In addition, the randomized design with the additional follow-up measurement will enable high level of evidence on the long-term effects of the resistance exercise intervention supplemented by nutritional optimization. Another advantage of this study represents the comprehensive evaluation of various health aspects using robust and meaningful outcome measures, with all tools showing good psychometric properties. The eligibility criterion are broad enabling an inclusion of various GvHD phenotypes. This is important for the external validity, especially in a heterogeneous disease. Further, there is a low threshold for participation, as participants will be actively approached via our study team during outpatient visit. Another strength represents the relatively large sample size. The heterogeneity of the disease manifestation calls for a multidisciplinary approach for patient management. Our multicomponent program was developed in accordance with (inter) national exercise and nutritional recommendations and in an interdisciplinary team. Adherence to exercise is a major challenge, particularly among immunosuppressed patients during Covid-19 pandemic. In order to reduce the risk of potential infections in training facility centers and increase adherence, we additionally offer online training supervised by exercise specialists. First findings show that online resistance training is safe and feasible, with a 5–10% higher attendance compared to in-person classes among prostate and breast cancer populations. Moreover, the study has shown similar effects of online group training compared to in-person training [104]. Based on these findings, we assume that online training is safe and feasible in GvHD patients too.

This study also has limitations that must be underlined. First, there is a lack of a usual care control group. Due to the high incidence of malnutrition among GvHD patients and the beneficial effect of nutritional support in cancer patients we considered it unethical to randomize persons to an untreated control group. Second, there is a risk for selection bias, as participants will be collected at a single site and only those willing and able to exercise will be included in the study. However, reasons for non-participation will be reported. Another limitation of the IRENE-G study include the lack of blinding of the study investigators and participants. The sample size has been calculated to be 112 participants in total, which is a challenge in a setting where patients’ health condition is highly variable, with fast deterioration up to death. Another disadvantage of using a single site represents the reduced external validity in contrast to multi-center trials. The exercise advice given in the control arm could be a potential confounder in determining the true effect of the experimental intervention, as participants might be encouraged to start exercising regularly. Nevertheless, given the strong evidence on exercise benefits, providing no advice was seen as unethical in this group.

## Conclusion

The IRENE-G study will bridge the research gap and contribute to our understanding of the value of exercise and nutritional endorsement in counteracting the negative consequences of GvHD and its treatment. To our knowledge, this will be the first randomized controlled trial that examines and compares the effects of a progressive moderate-to-high intensity resistance exercise supplemented by nutritional optimization against nutritional optimization only on physical performance in GvHD patients.

## Data Availability

Not applicable. Material developed to support this study is available from the corresponding author on reasonable request.

## Abbreviations

Allo HSCT: Allogeneic hematopoietic stem cell transplantation
GvHD: Graft-versus-host disease
SPPB: Short physical performance battery
mLSS: Modified 7-day Lee Chronic-versus-Host Diseases Symptom Scale
NRS 2002: Nutritional Risk Screening 2002
NCT: National Center for tumor diseases
VL: Vastus lateralis muscle
EORTC: European Organisation for Research and Treatment of Cancer
